# Ischemia-Modified Albumin, Lactate, and Combination for Predicting Mortality in Patients with Septic Shock in the Emergency Department

**DOI:** 10.3390/biomedicines12071421

**Published:** 2024-06-26

**Authors:** Bo-Yeong Jin, Sukyo Lee, Woosik Kim, Jong-Hak Park, Hanjin Cho, Sungwoo Moon, Sejoong Ahn

**Affiliations:** 1 Department of Biomedical Sciences, Seoul National University College of Medicine, Seoul 03080, Republic of Korea; boyeongjin0@gmail.com; 2 Department of Emergency Medicine, Korea University Ansan Hospital, Ansan-si 15355, Republic of Korea

**Keywords:** septic shock, ischemia modified albumin, lactic acid, prognosis, emergency department, risk stratification

## Abstract

Ischemia-modified albumin (IMA) is produced during ischemia and reactive oxygen species production. This study aimed to evaluate the association between IMA and mortality in a larger population and the prognostic value of the combination of IMA and lactate for predicting mortality in septic shock patients in the emergency department. This retrospective observational study included adult septic shock patients between October 2019 and December 2021. A multivariable Cox proportional hazards model was performed. IMA was significantly higher in the non-surviving group than in the surviving group (89.1 ± 7.2 vs. 83.8 ± 6.2 U/mL, *p* < 0.001). IMA was independently associated with 28-day mortality after adjustments (adjusted hazard ratio [aHR]: 1.075, 95% confidence interval [CI]: 1.016–1.138, *p* = 0.012). The area under the ROC curve (AUROC) of IMA was 0.712 (95% CI: 0.648–0.775, *p* < 0.001) and was comparable to that of lactate. The AUROC of the combination of IMA and lactate was 0.838 (95% CI: 0.786–0.889, *p* < 0.001). The group with both high lactate and high IMA levels showed an extremely high risk of mortality than other groups (86.1%; aHR 8.956, 95% CI 4.071–19.70, *p* < 0.001). The elevation of IMA was associated with mortality in septic shock patients. The combination of IMA and lactate can be a helpful tool for early risk stratification of septic shock patients.

## 1. Introduction

Sepsis is a life-threatening organ dysfunction caused by a dysregulated host response to infection [1]. Sepsis is a major global health problem with a high mortality [2,3]. Septic shock is the most severe form of sepsis, with a mortality rate of approximately 35–38% [3,4]. Early recognition, risk stratification, and comprehensive management are important to reduce mortality in patients with sepsis and septic shock, especially in emergency departments.

Several biomarkers have been proposed for predicting the outcomes of sepsis and septic shock. Lactate is a well-known biomarker of cellular metabolic dysfunction [3,5,6]. The elevation of lactate levels can be caused by inadequate tissue oxygen delivery, increased anaerobic metabolism, or increased lactate production [5,6,7]. Lactate is widely used to assess disease severity and predict mortality in critically ill patients, including those with sepsis and septic shock [5,6]. 

Ischemia-modified albumin (IMA) is an albumin with a decreased affinity for cobalt ions resulting from ischemia and the production of reactive oxygen species. [8] The ischemia and oxygen free radicals lead to modification of the N-terminal sequence of albumin, which results in decreased affinity for metals, including cobalt ions [9,10]. Furthermore, ischemia and oxygen-free radicals lead to the breakdown of tissues, plasma phospholipids, and triglycerides, resulting in increased plasma-free fatty acid concentrations. The binding of plasma-free fatty acids to albumin leads to a decreased affinity for cobalt ions [11]. The IMA increases within 10 min after the ischemic insult, remains elevated for 6–12 h, and normalizes after 12–24 h [12,13]. Therefore, the IMA can be used as a biomarker to evaluate ischemic insults. Although few studies have evaluated the prognostic value of IMA in patients with sepsis or severe sepsis, they only included a small number of patients with septic shock and did not evaluate the prognostic value of the combination of IMA and lactate levels [14,15]. 

In this study, we aimed to evaluate the association between IMA and mortality in a larger population of patients with septic shock. Furthermore, we evaluated the prognostic value of the combination of IMA and lactate levels in predicting mortality in patients with septic shock in the emergency department.

## 2. Materials and Methods

### 2.1. Study Design and Setting

This retrospective observational study was conducted at Korea University Ansan Hospital, the only tertiary academic teaching hospital in Ansan-si, where 700,000 residents live. Approximately 50,000 patients visit the emergency department of Korea University Ansan Hospital annually. This study was conducted in accordance with the principles of the Declaration of Helsinki. This study was approved by the Institutional Review Board of Korea University Ansan Hospital (2024AS0039). The requirement for informed consent was waived by the Institutional Review Board because of the observational design of this study.

### 2.2. Study Population

Adults (age ≥ 18 years) patients diagnosed with septic shock in the emergency department from October 2019 to December 2021 were included in this study. Patients with do-not-resuscitate (DNR) orders, unknown 28-day mortality, and missing IMA results were excluded. Patients with acute ischemic and embolic events and terminal malignancy were excluded because acute ischemic insults in acute myocardial infarction, ischemic stroke, embolism, massive hemorrhage, and chronic ischemic states in terminal malignancy could influence the IMA [14,15].

### 2.3. Definitions and Data Collection

Sepsis was defined as an acute increase in the total sequential organ failure assessment (SOFA) score ≥ 2 from baseline due to infection [1]. Septic shock was defined as a serum lactate level > 2 mmol/L and the requirement of vasopressors despite adequate fluid resuscitation to maintain a mean arterial pressure ≥ 65 mmHg. All patients were managed according to the Surviving Sepsis Campaign guidelines [3].

The following patient data were collected from the electronic medical records: age, sex, comorbidities, age-adjusted Charlson Comorbidity Index (CCI) [16], SOFA score, infection focus, initial vital signs, initial laboratory results, and outcomes.

### 2.4. Measurements

Peripheral venous blood was obtained before initial resuscitation using plastic vacutainer serum-separating tubes (Belliver Industrial Estate, Plymouth, UK). After separation of serum by centrifuging at 3500 rpm for 10 min, IMA was quantified using an albumin-cobalt binding assay (Medicalsystem Biotechnology Co., Ltd., Ningbo, China) with a Cobas c702 module analyzer (Roche Diagnostics GmbH, Mannheim, Germany), following the manufacturer’s instructions [15].

### 2.5. Outcome

The primary outcome was 28-day mortality. The secondary outcome was 90-day mortality.

### 2.6. Sample Size Calculations

Based on a subgroup analysis of 53 patients with septic shock in a previous study [15], the event rate was 26.4%, the adjusted hazard ratio (aHR) of IMA was 1.19, and the area under the receiver operating characteristic curve (AUROC) was 0.786. With a power of 0.9 and a significance level of 0.05, at least 63 patients are needed to evaluate the association between IMA and mortality in patients with septic shock, and at least 54 patients are needed to evaluate the prognostic value of IMA in patients with septic shock. However, to enhance the robustness and generalizability of our findings, we aimed to include a larger population of patients with septic shock.

### 2.7. Statistical Analysis

Continuous variables with normal distribution are expressed as means ± standard deviations and compared using the Student’s *t*-test. Continuous variables without normal distributions are expressed as medians [interquartile range] and compared using the Mann–Whitney U test. Categorical variables are expressed as numbers (percentages) and compared using the chi-square test or Fisher’s exact test.

The correlations between IMA and lactate, and IMA and SOFA scores were evaluated using Pearson’s correlation coefficient.

To evaluate the independent association between IMA and outcomes, a multivariable Cox proportional hazards model was used. Variables with a *p*-value < 0.1 in the univariable Cox proportional hazard model (Table S1) were entered into the multivariable Cox proportional hazard model.

Receiver operating characteristic (ROC) curves of the IMA, lactate, and combinations of IMA and lactate for outcomes were obtained. The AUROC was compared using the DeLong test. The optimal cut-off point was determined using the Youden index.

According to the optimal cutoff points of IMA and lactate, the study population was categorized into high lactate/high IMA, high lactate/low IMA, low lactate/high IMA, and low lactate/low IMA groups. Kaplan–Meier curves and log-rank tests were used to compare the outcomes between the groups. A multivariable Cox proportional hazards model was used to evaluate independent associations between the groups and outcomes.

Subgroup analysis according to infection focus (respiratory and non-respiratory origin) was performed.

Sensitivity analysis was performed after multiple imputations using Multivariate Imputation through Chained Equations (‘mice’ package) for patients with missing IMA results [17].

Statistical significance was set at *p* < 0.05. Bonferroni corrections were used in the post-hoc analysis. All statistical analyses were performed using R version 4.0.2 (R Foundation for Statistical Computing, Vienna, Austria).

## 3. Results

**Figure 1:**
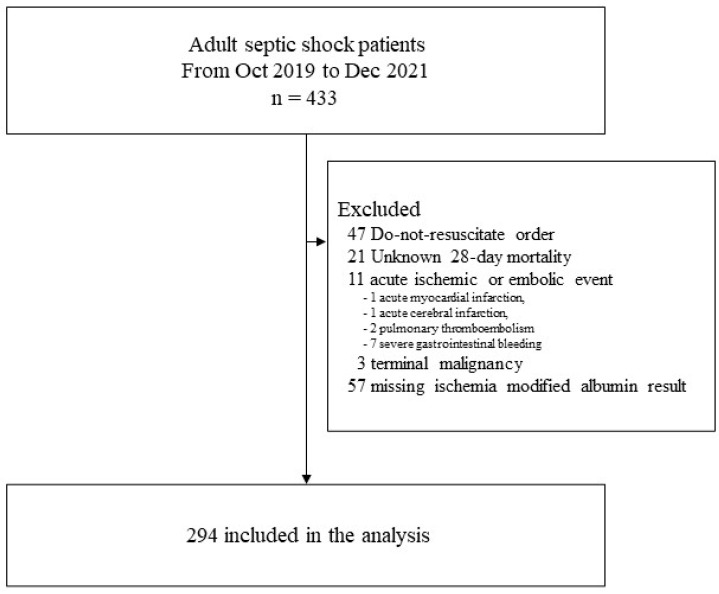
Flow chart of the study population.

We screened the data of 433 adults with septic shock between October 2019 and December 2021. Of these, 47 patients with DNR orders, 21 patients with unknown 28-day mortality, 11 patients with acute ischemic or embolic events (one acute myocardial infarction, one acute cerebral infarction, two pulmonary thromboembolisms, and seven severe gastrointestinal bleeding), 3 patients with terminal malignancy, and 57 patients with missing IMA results were excluded. Ultimately, 294 patients were included in the analysis (Figure 1). Mean age was 71.2 ± 14.1 years, 170 (57.8%) were men, and the mean SOFA score was 9.5 ± 2.7. The 28-day mortality rate was 31.3% (92/294) and the 90-day mortality rate was 41.0% (116/283). 

### 3.1. Baseline Characteristics

The baseline characteristics according to 28-day mortality are shown in Table 1. The initial SOFA score and age-adjusted CCI were significantly higher in the non-surviving group than in the surviving group. The infection focus of the respiratory origin was more frequent in the non-surviving group than in the surviving group. Body temperature and hemoglobin and albumin levels were significantly lower in the non-surviving group than in the surviving group. Lactate (9.0 [4.4–14.8] vs. 3.8 [2.9–6.1] mmol/L, *p* < 0.001) and IMA (89.1 ± 7.2 vs. 83.8 ± 6.2 U/mL, *p* < 0.001) were significantly higher in the non-surviving group than the surviving group. The baseline characteristics according to 90-day mortality (*n* = 283) were similar (Table S2).

### 3.2. Correlation Test

IMA was not correlated with lactate (R = 0.059, *p* = 0.314). IMA was not correlated with the SOFA score (R = 0.055, *p* = 0.348).

### 3.3. Multivariable Cox Proportional Hazards Model

IMA was independently associated with 28-day mortality after adjusting for infection focus, SOFA score, age-adjusted CCI, body temperature, hemoglobin, lactate, and albumin levels (aHR: 1.075, 95% confidence interval [CI]: 1.016–1.138, *p* = 0.012; Table 2). IMA was independently associated with 90-day mortality after adjustment (aHR 1.057, 95% CI 1.003–1.113, *p* = 0.038; Table S3).

The formula of the multivariable Cox proportional hazards model is shown below.

*h*(*t*) = *h*_0_(*t*) × exp(*β*_1_ × Infection focus + 1.108 × SOFA score + 1.116 × age-adjusted CCI + 0.990 × Body temperature + 1.055 × Hemoglobin + 1.104 × Lactate + 0.845 × Albumin + 1.075 × IMA) *β*_1_ for Infection focus: 0.789 (gastrointestinal infection), 0.330 (biliary infection), 0.507 (genitourinary infection), and 0.721 (others) (1)

### 3.4. Receiver Operating Characteristic Curve

**Figure 2:**
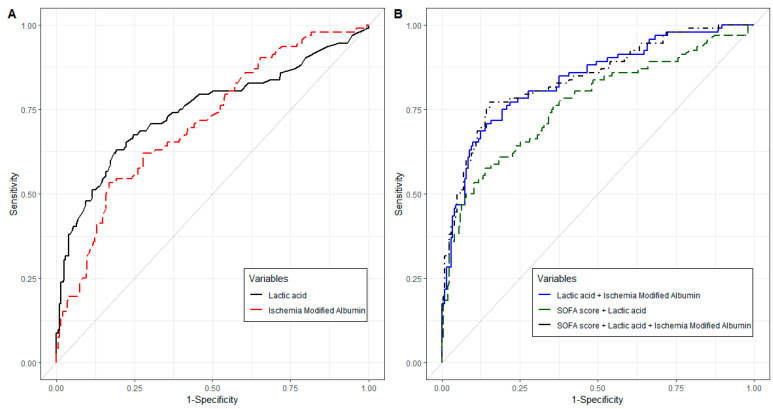
Receiver operating characteristic curve for 28-day mortality: (**A**) The black line shows the receiver operating characteristic curve of lactate and the red long-dashed line shows the receiver operating characteristic curve of IMA; (**B**) The blue line shows the receiver operating characteristic curve of a combination of lactate and IMA, the green long-dashed line shows the receiver operating characteristic curve of combination of SOFA score and lactate, and the black dot-dashed line shows the receiver operating characteristic curve of the combination of SOFA score, lactate, and IMA. Abbreviations: IMA, ischemia-modified albumin; SOFA, sequential organ failure assessment.

The AUROC of IMA for 28-day mortality was 0.712 (95% CI: 0.648–0.775, *p* < 0.001; Figure 2A and Table S4). The optimal cutoff of IMA was 89.1 U/mL with a sensitivity of 53.3% and a specificity of 83.2%.

The AUROC of lactate for 28-day mortality was 0.746 (95% CI: 0.679–0.813, *p* < 0.001; Figure 2A and Table S4). The optimal cutoff of lactate was 7.3 mmol/L with a sensitivity of 63.0% and a specificity of 80.7%. The AUROC was comparable between the IMA and lactate levels (*p* = 0.481; Table S5).

The AUROC of the combination of IMA and lactate levels for 28-day mortality was 0.838 (95% CI: 0.786–0.889, *p* < 0.001; Figure 2B and Table S4). The AUROC of the combination of IMA and lactate was comparable to that of the combination of IMA, lactate, and SOFA score (*p* = 0.576). The AUROC of the combination of IMA and lactate was significantly higher than that of lactate (*p* < 0.001), IMA (*p* < 0.001), or the combination of lactate and SOFA scores (*p* = 0.004; Table S5).

### 3.5. Analysis According to Group Categorized by Optimal Cutoffs of Lactate and Ischemia-Modified Albumin

The 28-day mortality rate was significantly higher in the high lactate/high IMA group than in the other groups (high lactate/high IMA, high lactate/low IMA, low lactate/high IMA, and low lactate/low IMA groups: 86.1% vs. 44.3% vs. 38.3% vs. 10.7%, respectively, *p* < 0.001; Table 3). The 28-day mortality was significantly different between the groups, except for the difference between the high lactate/low IMA and low lactate/high IMA groups in the post-hoc analysis (Table S6).

**Figure 3:**
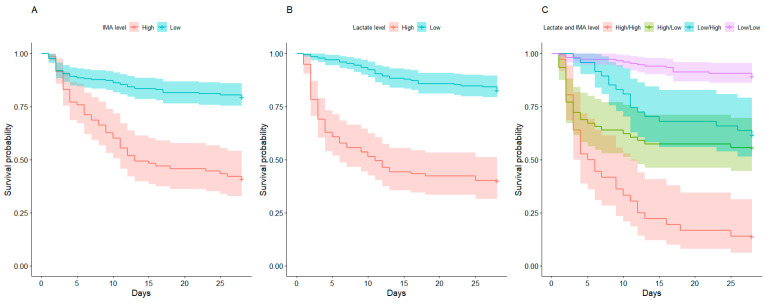
Kaplan–Meier curve according to groups: (**A**) Kaplan–Meier curve according to IMA level. The red line shows the survival probability of the high IMA group and the blue line shows the survival probability of the low IMA group; (**B**) Kaplan–Meier curve according to lactate level. The red line shows the survival probability of the high lactate group and the blue line shows the survival probability of the low lactate group; (**C**) Kaplan–Meier curve according to lactate and IMA level. The red line shows the survival probability of the high lactate/high IMA group, the green line shows the survival probability of the high lactate/low IMA group, the blue line shows the survival probability of the low lactate/high IMA group, and the purple line shows survival probability of low lactate/low IMA group. Abbreviations: IMA, ischemia modified albumin.

The Kaplan–Meier curve showed a significantly higher 28-day mortality in the high lactate/high IMA group than in the other groups (log-rank test, *p* < 0.001; Figure 3). The post-hoc analysis showed similar results.

In the multivariable Cox proportional hazards model, the high lactate/high IMA, high lactate/low IMA, and low lactate/high IMA groups were independently associated with higher 28-day mortality than the low lactate/low IMA group after adjustments (high lactate/high IMA group: aHR 8.956, 95% CI: 4.071–19.70, *p* < 0.001; high lactate/low IMA group: aHR 4.510, 95% CI: 2.357–8.630, *p* < 0.001; low lactate/high IMA group: aHR 2.757, 95% CI: 1.214–6.262, *p* = 0.015; Table S7).

### 3.6. Subgroup Analysis

In the subgroup analysis, according to infection focus (respiratory and non-respiratory origin), IMA was independently associated with 28-day mortality in both subgroups (aHR 1.089, 95% CI: 1.039–1.141, *p* < 0.001 in subgroup with infection focus of respiratory origin and aHR 1.133, 95% CI: 1.032–1.243, *p* = 0.009 in subgroup with infection focus of non-respiratory origin).

### 3.7. Sensitivity Analysis

In the sensitivity analysis after multiple imputations for missing IMA results, IMA was independently associated with 28-day mortality after adjusting for the aforementioned covariables (aHR 1.083, 95% CI: 1.051–1.117, *p* < 0.001).

## 4. Discussion

In patients with septic shock, IMA was independently associated with mortality. The AUROC of IMA was comparable to that of lactate levels. The combination of lactate and IMA resulted in a better prediction of mortality than lactate alone, IMA alone, or a combination of the SOFA score and lactate. When patients with septic shock were categorized according to the optimal cut-offs of lactate and IMA, the high lactate/high IMA group showed an extremely high risk of mortality, the high lactate/low IMA and low lactate/high IMA groups showed an intermediate risk of mortality, and the low lactate/low IMA group showed a low risk of mortality.

Previous studies that evaluated the prognostic value of IMA in patients with sepsis or severe sepsis reported an independent association between IMA and short-term mortality and a similar AUROC of IMA for short-term mortality, which supports our results [14,15]. However, these previous studies included only 117 and 124 patients with sepsis; of these, 34 and 53 patients with septic shock were included, respectively [14,15]. Another previous study reported the poor prognostic value of IMA; however, this study included a small number of patients with sepsis and did not adjust for important covariables such as lactate level, SOFA score, or comorbidities [18]. Our study included a large number of patients with septic shock (n = 294), leading to more generalized results, particularly for patients with septic shock. Therefore, the IMA can be a good prognostic marker for the early prediction of mortality in patients with septic shock.

The Kaplan–Meier curve according to lactate and IMA levels showed different patterns of increased mortality in specific periods. The high-lactate group showed a sharp decline in survival probability within a few days of septic shock, whereas the high-IMA group showed a gradual decline in survival probability for up to 14 days after septic shock. This pattern of the Kaplan–Meier curve of IMA was similar to that reported in previous studies [14,15]. When patients were categorized according to lactate and IMA levels, high lactate and IMA patterns remained similar in the subgroups.

The difference in the survival pattern, the result showing no correlation between IMA and lactate, and the independent association of IMA after adjustments, including lactate, can be explained by the different mechanisms of lactate and IMA production. Inadequate oxygen delivery occurs in patients with septic shock [19], resulting in anaerobic metabolism that produces lactate. Furthermore, mitochondrial dysfunction in sepsis leads to increased anaerobic metabolism, despite adequate oxygen delivery [7]. As the time from inadequate oxygen delivery to death is short [7], the high lactate group had a high risk of death within a few days of septic shock.

Sepsis causes an imbalance between reactive oxygen species and antioxidants [20]. Alterations in the endothelium, neutrophils, macrophages, and mitochondrial signaling pathways during sepsis significantly induce oxidative stress [20]. Ischemic insult is associated with oxidative stress, and the production of reactive oxygen species affects albumin and produces IMA [8,9,10,11]. The high IMA group experienced high oxidative stress, which is associated with sepsis-induced endothelial dysfunction that causes microcirculatory failure and multiorgan failure [19]. This organ dysfunction may contribute to the continuous risk of death and lead to a gradual decline in survival probability for up to 14 days after septic shock. Therefore, in addition to lactate levels, IMA can be used to predict the prognosis of patients with septic shock.

Notably, patients with septic shock with both high lactate and high IMA showed an extremely high risk of mortality, whereas those with any single increase in lactate or IMA showed an intermediate risk of mortality, and those with both low lactate and low IMA showed an extremely low risk of mortality. These results were maintained even after adjusting for other variables. This might be due to the independent mechanisms of lactate and IMA production, as described above, which may have synergistic effects on predictive power. Furthermore, the combination of lactate and IMA showed good performance in predicting mortality in patients with septic shock, which is comparable to the combination of SOFA score, lactate, and IMA, and is better than the combination of the SOFA score and lactate. Therefore, any elevation of lactate or IMA and the combination of lactate and IMA would be helpful in the early risk stratification of patients with septic shock in the early stages of management. Moreover, physicians must be aware of the extremely high mortality risk of septic shock patients with both elevated lactate and IMA levels because significantly insufficient oxygen supply and substantial ischemic insult occur in these patients.

As the production of IMA is caused by ischemia and reactive oxygen species and is not infection-specific, IMA can be another representative marker of patient severity. IMA is associated with poor prognosis in critically ill diseases, such as aortic dissection [21,22], traumatic brain injury [23], and cardiac arrest [24,25]. It would be more helpful if lactate and IMA levels were used together for risk stratification in critically ill patients; however, this requires further study.

Although both the SOFA score and IMA were higher in the non-surviving group than in the surviving group, IMA was not correlated with the SOFA score. Additionally, IMA was independently associated with mortality even after adjusting for the SOFA score. This suggests that IMA could be an additional prognostic marker besides the SOFA score. However, this study only assessed SOFA scores on the first day of admission. Therefore, it remains unclear whether IMA is correlated with SOFA score after a few days of admission or with the worst SOFA score during the hospital stay. Further research is needed to explore these relationships.

The strength of our study is that we included a large number of patients with septic shock compared to previous studies [14,15,18]. Furthermore, we evaluated the correlation between IMA and lactate and the independent association of IMA with mortality after adjusting for important covariables, such as lactate, SOFA score, and comorbidities, and directly compared the performance of lactate and IMA in predicting mortality. In addition to 28-day mortality, we first evaluated 90-day mortality and found that IMA was associated with long-term mortality. Moreover, to the best of our knowledge, this is the first study to evaluate the usefulness of the combination of lactate and IMA levels in predicting the prognosis of patients with septic shock.

This study had some limitations. First, as this was an observational study, there could have been missed covariables. In addition, we could only find associations and not causalities. Second, this study was conducted at a single center. These results cannot be generalized to the entire population. Third, the IMA can differ according to the measurement protocols in each laboratory setting. The results and measurements must be validated before they can be applied. Fourth, some patients were excluded owing to missing IMA results. However, similar results were found in the sensitivity analysis after multiple imputations for missing IMA results.

## 5. Conclusions

In patients with septic shock, the elevation of ischemia-modified albumin was associated with mortality. The combination of ischemia-modified albumin and lactate showed good performance in predicting short-term mortality and can be a helpful tool for the early risk stratification of patients with septic shock.

## Figures and Tables

**Table 1:** Baseline characteristics according to 28-day mortality.

Variables	Survived on Day 28 (*n* = 202)	Died (*n* = 92)	*p*-Value
Sex			0.558
Men	114 (56.4%)	56 (60.9%)	
Women	88 (43.6%)	36 (39.1%)	
Age (years)	74 [62–81]	76 [64–83]	0.378
Infection focus			<0.001
Respiratory	58 (28.7%)	52 (56.5%)	
Gastrointestinal	28 (13.9%)	10 (10.9%)	
Biliary	45 (22.3%)	7 (7.6%)	
Genitourinary	58 (28.7%)	15 (16.3%)	
Others	13 (6.4%)	8 (8.7%)	
SOFA score	9 [7–10]	11 [9–12]	<0.001
Comorbidities			
Age-adjusted Charlson Comorbidity Index	4.4 ± 2.3	5.0 ± 2.6	0.050
Diabetes mellitus	80 (39.6%)	39 (42.4%)	0.746
Hypertension	123 (60.9%)	48 (52.2%)	0.201
Cardiac disease	29 (14.4%)	8 (8.7%)	0.243
Liver disease	9 (4.5%)	6 (6.5%)	0.645
Chronic kidney disease	19 (9.4%)	14 (15.2%)	0.206
Lung disease	7 (3.5%)	5 (5.4%)	0.636
Stroke	32 (15.8%)	12 (13.0%)	0.655
Malignancy	39 (19.3%)	25 (27.2%)	0.173
Initial vital signs			
Systolic blood pressure (mmHg)	106 [91–123]	102 [87.5–130.5]	0.432
Diastolic blood pressure (mmHg)	63 [55–75]	61 [52–76]	0.339
Heart rate (/min)	104 [90–122]	110.5 [88–128]	0.231
Respiratory rate (/min)	20 [18–23]	22 [16–28]	0.471
Body temperature	37.3 ± 1.3	36.6 ± 1.3	<0.001
Laboratory results			
Hemoglobin (g/dL)	11.9 ± 2.6	11.1 ± 3.0	0.034
White blood cell (*10^3^/μL)	12.0 ± 8.4	10.6 ± 8.8	0.178
Platelets (*10^3^/μL)	156 [108–233]	168 [82–253]	0.872
Creatinine (mg/dL)	1.4 [0.9–2.0]	1.6 [1.0–2.4]	0.194
Total bilirubin (mg/dL)	0.8 [0.5–1.4]	0.9 [0.4–1.8]	0.357
CRP (mg/dL)	9.4 [3.2–20.8]	10.8 [6.7–19.8]	0.303
Procalcitonin (ng/mL)	6.4 [1.2–26.0]	4.2 [1.4–17.0]	0.415
Lactate (mmol/L)	3.8 [2.9–6.1]	9.0 [4.4–14.8]	<0.001
Albumin (g/dL)	3.5 [3.1–3.9]	3.0 [2.6–3.3]	<0.001
Ischemia-modified albumin (U/mL)	83.8 ± 6.2	89.1 ± 7.2	<0.001

Data are presented as median [interquartile range], mean ± standard deviation, or number (%), as appropriate. Abbreviations: SOFA, sequential organ failure assessment; CRP, C-reactive protein.

**Table 2:** Multivariable Cox proportional hazards model for 28-day mortality.

Variables	Adjusted Hazard Ratio	95% Confidence Interval	*p*-Value
Infection focus			
Respiratory	Reference		
Gastrointestinal	0.789	0.391–1.591	0.507
Biliary	0.330	0.147–0.742	0.007
Genitourinary	0.507	0.277–0.929	0.028
Others	0.721	0.328–1.583	0.414
SOFA score	1.108	1.009–1.216	0.031
Age-adjusted Charlson Comorbidity Index	1.116	1.018–1.224	0.019
Body temperature	0.990	0.830–1.180	0.907
Hemoglobin (g/dL)	1.055	0.966–1.152	0.233
Lactate (mmol/L)	1.104	1.071–1.139	<0.001
Albumin (g/dL)	0.845	0.412–1.734	0.646
Ischemia-modified albumin (U/mL)	1.075	1.016–1.138	0.012

Abbreviations: SOFA, sequential organ failure assessment.

**Table 3:** The 28-day mortality according to groups by optimal cutoffs.

	High Lactate (*n* = 97)	Low Lactate (*n* = 197)
High ischemia-modified albumin (*n* = 83)	31/36 (86.1%)	18/47 (38.3%)
Low ischemia-modified albumin (*n* = 211)	27/61 (44.3%)	16/150 (10.7%)

## Data Availability

The datasets used and/or analyzed in the current study are available from the corresponding author upon reasonable request.

## Supplementary Materials

The following supporting information can be downloaded at: https://www.mdpi.com/article/10.3390/biomedicines12071421/s1, Table S1: Univariable Cox proportional hazard model; Table S2: Baseline characteristics according to 90-day mortality; Table S3: Multivariable Cox proportional hazard model for 90-day mortality; Table S4: Result of receiver operation characteristic curve for 28-day mortality; Table S5: Pairwise comparison of area under the receiver operating characteristic curves (AUROCs); Table S6: Pairwise comparison between groups; Table S7: Multivariable Cox proportional hazard model using groups according to optimal cutoffs.
