# Supplementary material for: Ischemia-Modified Albumin, Lactate, and Combination for Predicting Mortality in Patients with Septic Shock in the Emergency Department
Source: Biomedicines. 2024 Jun 26;12(7):1421. doi: 10.3390/biomedicines12071421 (PMC11274077; doi:10.3390/biomedicines12071421)
Supplement: Supplementary file 1 [file biomedicines-12-01421-s001.zip › biomedicines-3060702-supplementary.pdf]

Table S1. Univariable Cox proportional hazard model.

	HR	95% CI	p-value
Sex (reference: men)	1.172	0.771–1.781	0.458
Age (years)	1.005	0.990–1.020	0.532
Infection focus			
Respiratory	reference		
Gastrointestinal	0.500	0.254–0.984	0.045
Biliary	0.237	0.108–0.521	<0.001
Genitourinary	0.670	0.208–0.657	0.001
Others	0.719	0.342–1.514	0.385
SOFA score	1.246	1.159–1.340	<0.001
**Comorbidities**			
Age-adjusted Charlson Comorbidity Index	1.087	1.002–1.180	0.045
Diabetes mellitus	1.103	0.729–1.668	0.643
Hypertension	0.741	0.492–1.116	0.151
Cardiac disease	0.609	0.295–1.259	0.181
Liver disease	1.464	0.640–3.351	0.367
Chronic kidney disease	1.510	0.855–2.667	0.156
Lung disease	1.542	0.626–3.798	0.347
Stroke	0.800	0.436–1.468	0.472
Malignancy	1.424	0.899–2.255	0.132
**Initial vital signs**			
Systolic blood pressure	0.997	0.990–1.004	0.377
Diastolic blood pressure	0.994	0.982–1.005	0.279
Heart rate	1.004	0.997–1.011	0.287
Respiratory rate	1.004	0.981–1.028	0.755
Body temperature	0.681	0.574–0.807	<0.001
**Laboratory results**			
Hemoglobin	0.921	0.855–0.993	0.032
White blood cell	0.979	0.952–1.006	0.129
Platelets	1.000	0.998–1.002	0.993
Creatinine	1.081	0.959–1.218	0.204
Total bilirubin	1.085	1.017–1.158	0.013
CRP	1.004	0.845–1.023	0.693
Procalcitonin	0.996	0.989–1.004	0.315
Lactate	1.139	1.112–1.166	<0.001
Albumin	0.245	0.165–0.364	<0.001
Ischemia modified albumin	1.087	1.057–1.117	<0.001

Abbreviations: HR, hazard ratio; CI, confidence interval; SOFA, sequential organ failure assessment

Table S2. Baseline characteristics according to 90-day mortality.

	Survived at day 90 (N=167)	Died (N=116)	p-value
Sex			1.000
Men	96 (57.5%)	66 (56.9%)	
Women	71 (42.5%)	50 (43.1%)	
Age (years)	72 [61.5–81]	77 [66.5–83]	0.059
Infection focus			<0.001
Respiratory	45 (26.9%)	60 (51.7%)	
Gastrointestinal	27 (16.2%)	11 (9.5%)	
Biliary	38 (22.8%)	11 (9.5%)	
Genitourinary	48 (28.7%)	23 (19.8%)	
Others	9 (5.4%)	11 (9.5%)	
SOFA score	8 [7–10]	11 [8–12]	<0.001
**Comorbidities**			
Age-adjusted Charlson Comorbidity Index	4.2 ± 2.3	5.1 ± 2.5	0.001
Diabetes mellitus	64 (38.3%)	51 (44.0%)	0.408
Hypertension	102 (61.1%)	63 (54.3%)	0.311
Cardiac disease	21 (12.6%)	14 (12.1%)	1.000
Liver disease	7 (4.2%)	7 (6.0%)	0.671
Chronic kidney disease	11 (6.6%)	20 (17.2%)	0.009
Lung disease	7 (4.2%)	5 (4.3%)	1.000
Stroke	24 (14.4%)	17 (14.7%)	1.000
Malignancy	32 (19.2%)	31 (26.7%)	0.174
**Initial vital signs**			
Systolic blood pressure (mmHg)	106 [92–123.5]	102.5 [87.5–128]	0.214
Diastolic blood pressure (mmHg)	64 [55–76]	60.5 [52–73]	0.123
Heart rate (/min)	104 [90–120]	110 [88–127.5]	0.432
Respiratory rate (/min)	20 [18–23]	21.5 [16–26]	0.753
Body temperature (°C)	37.4 ± 1.3	36.7 ± 1.2	<0.001
**Laboratory results**			
Hemoglobin (g/dL)	12.0 ± 2.6	11.0 ± 2.9	0.004
White blood cell ($*10^3/\mu L$)	12.1 ± 8.6	10.9 ± 8.5	0.252
Platelets ($*10^3/\mu L$)	151 [107–222]	181.5 [91–269]	0.322
Creatinine (mg/dL)	1.4 [0.9–1.9]	1.6 [1.0–2.5]	0.098
Total bilirubin (mg/dL)	0.8 [0.5–1.4]	0.9 [0.5–1.6]	0.552
CRP (mg/dL)	9.2 [3.4–20.1]	10.7 [6.4–21.2]	0.183
Procalcitonin (ng/mL)	7.3 [1.0–26.3]	4.0 [1.5–20.0]	0.543

Lactate (mmol/L)	3.9 [2.9–5.6]	7.8 [3.7–13.2]	<0.001
Albumin (g/dL)	3.5 [3.1–4.0]	3.0 [2.6–3.5]	<0.001
Ischemia modified albumin (U/mL)	83.5 ± 6.3	88.3 ± 7.2	<0.001

Data are presented as median [interquartile range], mean ± standard deviation, or number (%), as appropriate.

Abbreviations: SOFA, sequential organ failure assessment; CRP, C-reactive protein

Table S3. Multivariable Cox proportional hazard model for 90-day mortality.

	aHR	95% CI	p-value
Infection focus			
Respiratory	Reference		
Gastrointestinal	0.631	0.325–1.224	0.173
Biliary	0.408	0.210–0.791	0.008
Genitourinary	0.550	0.330–0.914	0.021
Others	0.868	0.440–1.711	0.682
SOFA score	1.090	1.002–1.184	0.044
Age-adjusted Charlson Comorbidity Index	1.121	1.034–1.214	0.005
Body temperature	0.957	0.818–1.120	0.587
Hemoglobin	1.036	0.956–1.122	0.393
Lactate	1.097	1.066–1.130	<0.001
Albumin	0.739	0.380–1.439	0.374
Ischemia modified albumin	1.057	1.003–1.113	0.038

Abbreviations: aHR, adjusted hazard ratio; CI, confidence interval; SOFA, sequential organ failure assessment

Table S4. Result of receiver operation characteristic curve for 28-day mortality.

	AUROC	95% confidence interval
Lactate	0.746	0.679–0.813
Ischemia modified albumin	0.712	0.648–0.775
Lactate + Ischemia modified albumin	0.838	0.786–0.889
SOFA score + Lactate	0.766	0.703–0.830
SOFA score + Lactate + Ischemia modified albumin	0.841	0.790–0.893

Abbreviations: AUROC, area under receiver operation characteristic curve; SOFA, sequential organ failure assessment

Table S5. Pairwise comparison of area under the receiver operating characteristic curves (AUROCs).

Comparison	p-value*
Lactate vs. Ischemia modified albumin	0.481
Lactate vs. Lactate + Ischemia modified albumin	<0.001
Lactate vs. SOFA score + Lactate	0.170
Lactate vs. SOFA score+ Lactate + Ischemia modified albumin	<0.001
Ischemia modified albumin vs. Lactate + Ischemia modified albumin	<0.001
Ischemia modified albumin vs. SOFA score + Lactate	0.247
Ischemia modified albumin vs. SOFA score+ Lactate + Ischemia modified albumin	<0.001
Lactate + Ischemia modified albumin vs. SOFA score + Lactate	0.004
Lactate + Ischemia modified albumin vs. SOFA score + Lactate + Ischemia modified albumin	0.576
SOFA score + Lactate vs. SOFA score + Lactate + Ischemia modified albumin	<0.001

*p-value<0.005 is considered significant after Bonferroni corrections

Abbreviations: SOFA, sequential organ failure assessment

Table S6. Pairwise comparison between groups.

Comparison (Lactate/Ischemia modified albumin level)	p-value*
High/High vs. High/Low	<0.001
High/High vs. Low/High	<0.001
High/High vs. Low/Low	<0.001
High/Low vs. Low/High	1.000
High/Low vs. Low/Low	<0.001
Low/High vs. Low/Low	<0.001

*p-value<0.0083 is considered significant after Bonferroni corrections

Table S7. Multivariable Cox proportional hazard model using groups according to optimal cutoffs.

Lactate/Ischemia modified albumin level	aHR	95% CI	p-value
Low/Low	Reference		
Low/High	2.757	1.214–6.262	0.015
High/Low	4.510	2.357–8.630	<0.001
High/High	8.956	4.071–19.70	<0.001

Adjusted for infection focus, SOFA score, Age-adjusted Charlson Comorbidity Index, body temperature, hemoglobin, and albumin

Abbreviations: aHR, adjusted hazard ratio; CI, confidence interval; SOFA, sequential organ failure assessment
